# Fast empirical Bayesian LASSO for multiple quantitative trait locus mapping

**DOI:** 10.1186/1471-2105-12-211

**Published:** 2011-05-26

**Authors:** Xiaodong Cai, Anhui Huang, Shizhong Xu

**Affiliations:** 1 Department of Electrical and Computer Engineering, University of Miami, Coral Gables, FL 33146, USA; 2 Department of Botany and Plant Sciences, University of California, Riverside, CA 92521, USA

## Abstract

**Background:**

The Bayesian shrinkage technique has been applied to multiple quantitative trait loci (QTLs) mapping to estimate the genetic effects of QTLs on quantitative traits from a very large set of possible effects including the main and epistatic effects of QTLs. Although the recently developed empirical Bayes (EB) method significantly reduced computation comparing with the fully Bayesian approach, its speed and accuracy are limited by the fact that numerical optimization is required to estimate the variance components in the QTL model.

**Results:**

We developed a fast empirical Bayesian LASSO (EBLASSO) method for multiple QTL mapping. The fact that the EBLASSO can estimate the variance components in a closed form along with other algorithmic techniques render the EBLASSO method more efficient and accurate. Comparing with the EB method, our simulation study demonstrated that the EBLASSO method could substantially improve the computational speed and detect more QTL effects without increasing the false positive rate. Particularly, the EBLASSO algorithm running on a personal computer could easily handle a linear QTL model with more than 100,000 variables in our simulation study. Real data analysis also demonstrated that the EBLASSO method detected more reasonable effects than the EB method. Comparing with the LASSO, our simulation showed that the current version of the EBLASSO implemented in Matlab had similar speed as the LASSO implemented in Fortran, and that the EBLASSO detected the same number of true effects as the LASSO but a much smaller number of false positive effects.

**Conclusions:**

The EBLASSO method can handle a large number of effects possibly including both the main and epistatic QTL effects, environmental effects and the effects of gene-environment interactions. It will be a very useful tool for multiple QTL mapping.

## Background

Quantitative traits are usually controlled by multiple quantitative trait loci (QTLs) and environmental factors. Interactions among QTLs or between genes and environmental factors make a substantial contribution to variation in complex traits [1]. The goal of QTL mapping is to infer genomic loci that are associated with the trait and to estimate the genetic effects of these loci including their main effects and gene-gene (epistasis) and gene-environment (*G *× *E*) interactions. Due to the physical linkage of and/or epistatic interactions among multiple QTLs, it is highly desirable to analyze a large number of loci simultaneously. Since hundreds or thousands of genomic loci or markers are usually genotyped and involved in QTL mapping studies, including all these markers and their possible interactions in a model leads to a huge number of model variables, typically much larger than the sample size. Two general techniques often employed to handle such oversaturated models are variable selection and shrinkage.

Variable selection attempts to identify a subset of all possible genetic effects that best explain the phenotypic variation, typically using a stepwise search procedure in conjunction with a selection criterion such as the Bayesian information criterion (BIC) [2]. On the other hand, a shrinkage method includes all variables in the model but uses a penalty function of the variables or appropriate prior distributions on the variables to shrink most non-effect variables toward zero. Early shrinkage methods include ridge regression [3] and the least absolute shrinkage and selection operator (LASSO) [4]. More recently, Bayesian shrinkage method [5] has received considerable attention and been applied to multiple QTL mapping [6-10]. All these works employ Markov chain Monte Carlo (MCMC) simulation to fit the Bayesian model and provide comprehensive information about the model drawing from the posterior distribution of the model variables. Despite the advances in the development of the MCMC simulation algorithms [11], MCMC simulations are computationally intensive and time consuming.

In order to reduce the computational burden of the fully Bayesian approach relying on MCMC simulation, one of the authors of this paper developed an empirical Bayes (EB) method [12] that uses a properly chosen prior distribution for the model variables to shrink variables toward zero. It was demonstrated that the EB method can handle a large number of model variables simultaneously. More recently, the EB method has been extended to handle classification predictor variables [13]. Although the EB method [12] requires much less computation comparing to the fully Bayesian approach, its efficiency is limited by the fact that a numerical optimization algorithm such as the simplex algorithm [14] is needed to estimate the variance components. On the other hand, a very efficient EB method, named relevance vector machine (RVM), for learning a linear model was developed in the machine learning community [15,16]. The RVM can estimate the variance components in a closed form, which along with other algorithmic techniques make it a very fast algorithm. The RVM assumes a uniform prior distribution for the variance components. Although this choice of the prior distribution gets rid of any hyperparameters to be pre-specified, it lacks the flexibility of adjusting the degree of shrinkage needed for analyzing a specific data set. Particularly, its uniform prior distribution may not provide enough shrinkage in multiple QTL mapping that includes a very large number of possible effects, often resulting in a large number of false effects [13].

In this paper, capitalizing on the idea of RVM, we developed a fast empirical Bayesian LASSO (EBLASSO) algorithm based on the Bayesian LASSO model [10,17] with an exponential prior distribution for the variance components in contrast to the inverse chi-square distribution for the variance components used by the EB method [12]. Simulation studies demonstrate that our EBLASSO method can provide a speed up to orders of magnitude faster than the EB method and can detect more true QTL effects without increasing the false positive rate. Real data analysis also show that the EBLASSO method is able to detect some effects when the EB method fails.

## Methods

### Linear model of multiple QTLs

Let *y_i _*be the phenotypic value of a quantitative trait of the *i*th individual in a mapping population. Suppose we observe *y_i_*, *i *= 1, ⋯, *n *of *n *individuals and collect them into a vector **y **= [*y*_1_, *y*_2_, ⋯, *y_n_*]*^T^*. Considering environmental effects, main and epistatic effects of all markers and gene-environment (*G *× *E*) interactions, we have the following linear regression model for **y**:(1)

where *μ *is the population mean, vectors *β_E _*and *β_G _*represent the environmental effects and the main effects of all markers, respectively, vectors *β_GG _*and *β_GE _*capture the epistatic effects and the *G *× *E *interactions, respectively, **X***_E_*, **X***_G_*, **X***_GG _*and **X***_GE _*are the corresponding design matrices of different effects, and **e **is the residual error that follows a normal distribution with zero-mean and covariance . Throughout the paper we use **I **to denote an identity matrix whose size can be clearly identified from the context.

The design matrix **X***_G _*depends on a specific genetic model. We adopt the widely used Cockerham genetic model as also used by [18] in their generalized linear model for multiple QTL mapping. For a back-cross design, the Cockerham model defines the values of the main effect of a marker as -0.5 and 0.5 for two genotypes at the marker. For an intercross (F_2_) design, there are two possible main effects named additive and dominance effects. The Cockerham model defines the values of the additive effect as -1, 0 and 1 for the three genotypes and the values of the dominance effect as -0.5 and 0.5 for homozygotes and heterozygotes, respectively. The columns of the design matrix **X***_GG _*are obtained as the element-wise product of any two different columns of **X***_G_*, and similarly the columns of **X***_GE _*are constructed as the element-wise product of any pair of columns from **X***_E _*and **X***_G_*.

Defining , **X **= [**X***_E_*, **X***_G_*, **X***_GG_*, **X***_GE_*], we can write (1) in a more compact form:(2)

Suppose that there are *p *environmental covariates and *q *markers whose main effects are additive, then the size of matrix **X **is *n *× *k *where *k *= *p *+ *q*(*q *+ 1)/2 + *pq*. Typically, we have *k *≫ *n*. If dominance effects of the markers are considered, *k *is even larger. Our goal is to estimate all possible environmental and genetical effects on **y **manifested in the regression coefficients ***β***, which is a challenging problem because *k *≫ *n*. However, we would expect that most elements of ***β ***are zeros and thus we have a sparse linear model. Taking into account this sparsity, we will adopt the Bayesian LASSO model [10] where appropriate prior distributions are assigned to the elements of ***β ***as described in the next section.

### Prior and posterior distributions

The unknown parameters in model (2) are, *μ * and ***β***. While our main concern is ***β***, parameters *μ *and  need to be estimated so that we can infer ***β***. To this end, we assign a noninformative uniform prior *μ *to and , i.e., *p*(*μ*) ∝ 1 and *p*() ∝ 1. Following the Bayesian LASSO model [10], we assume a three-level hierarchical model for ***β***. Let us denote the elements of ***β ***as *β_i_*, *i *= 1, 2, ⋯, *k*. At the first level, *β_i_*, *i *= 1, 2, ⋯, *k *follow independent normal distributions with mean zero and unknown variance . At the second level, , follow independent exponential distribution with a common parameter . For a given *λ*, the distribution of *β_i _*is found to be the Laplace distribution: , which is known to encourage the shrinkage of *β_i _*toward zero [4]. However, the degree of shrinkage strongly depends on the value of *λ*. To alleviate the problem of choosing an inappropriate value for *λ*, we add another level to the hierarchical model at which we assign a conjugate Gamma prior Gamma(*a, b*) with a shape parameter *a *> 0 and an inverse scale parameter *b *> 0 to the parameter *λ*. As discussed in [10], we can pre-specify appropriate values for *a *and *b *so that the Gamma prior for *λ *is essentially noninformative.

Let us define vector . The joint posterior distribution of all the parameters (*μ*, , ***β***, ***σ***^2^, *λ*) can be easily found [10]. In principle, MCMC simulation can be employed to draw samples from the posterior distribution for each parameter. However, since the number of parameters 2*k *+ 3 in our model can be very large, the fully Bayesian approach based on MCMC sampling requires a prohibitive computational cost. To avoid this problem, Xu developed an empirical Bayes method for inferring ***β ***[12]. Our goal here is to develop a much faster and more accurate empirical Bayes method that can easily handle tens of thousands of variables.

### Maximum a posteriori estimation of variance components

Similar to the EB method of [12], our EBLASSO first estimates ***σ***^2^,  and *μ*, and then finds the posterior distribution of ***β ***based on the estimated parameters. Since *λ *is a parameter that we do not want to estimate, we can find the prior distribution of  independent of *λ *as follows(3)

The posterior distribution of *μ*, ***β***, ***σ***^2 ^and  is given by(4)

The marginal posterior distribution of *μ*, ***σ***^2 ^and  can then be written as(5)

Let us define the precision of *β_i _*as , *i *= 1, 2, ..., *k *and let ***α ***= [*α*_1_, *α*_2_, ⋯, *α_k_*]. It turns out to be more convenient to estimate ***α ***rather than ***σ***^2 ^as will be shown shortly. Let us collect all parameters that need to be estimated as ***θ ***= (*μ*, *σ*_0_, ***α***). The log marginal posterior distribution of ***θ ***can be found from (5) as follows(6)

where  is the covariance matrix of **y **with a given ***α***.

Similar to the EB method [12] and the RVM [15,16], we will iteratively estimate each parameter by maximizing the log marginal posterior distribution *L*(***θ***) with the other parameters being fixed. Since it is not difficult to find the optimal *μ *and  in each iteration as shown in [12,15], we will give the expressions for *μ *and  later but focus on the estimation of ***α ***now. Let us define  and . Then following the derivations in [16], we can write *L*(***θ***) in (6) as *L*(***θ***) = *L*(***θ***_-*i*_) + *L*(*α_i_*), where *L*(***θ***_-*i*_) does not depend on *α_i _*and *L*(*α_i_*) is given by(7)

with  and . If we assume *a *> -1.5 and *b *> 0, we prove in the additional file that *L*(*α_i_*) has a unique global maximum and that the optimal *α_i _*maximizing *L*(*α_i_*) is given by(8)

where we have defined , , ,  and . Note that the Gamma distribution requires that *a *> 0 and *b *> 0 as we mentioned earlier. When -1.5 <*a *≤ 0 as assumed here, we essentially use an improper distribution. In the additional file, we show that this improper distribution appears appropriate for getting a point estimation of *α_i _*given in (8). It turns out that negative values of *a *give one more degree of freedom to adjust the shrinkage as will be demonstrated later in the Results section. Moreover, if *a *= -1, the last term in the right hand side of (7) disappears. In this case, we essentially use a noninformative uniform prior. Then it is not difficult to verify that (8) gives the optimal *α_i _*derived in [16]:(9)

Note that α* in (8) and (9) depends on other unknown parameters through *s_i _*and *q_i_*, and thus, *α_i _*will be estimated iteratively as detailed in the EBLASSO algorithm described in the next section. Comparing with the EB method [12], our method finds each *α_i _*(and equivalently ) in a closed form, whereas the EB method generally needs to employ a numerical optimization algorithm to find each . Therefore, our method not only saves much computation but also gives more accurate estimate of each *α_i_*. Moreover, exploiting the similar techniques used in the RVM [16], we can further increase computational efficiency as described in the ensuing section.

### Fast Empirical Bayesian LASSO algorithm

Note that when *α_i _*= *∞*, we have *β_i _*= 0. Therefore, in each iteration, we can construct a reduced model of (2) that includes only nonzero *β_i_*s and the corresponding columns of **X**. Let **x***_i _*be the *i*th column of **X**. If *α_i _*= *∞ *in the previous iteration but  is finite in the current iteration, then we add **x***_i _*to the model and set ; if *α_i _*is finite in the previous iteration but  in the current iteration, we delete **x***_i _*from the model and set *α_i _*= *∞*; if both *α_i _*and  are finite, we retain **x***_i _*in the model and update *α_i _*as . This can be done for all *i*s in a pre-specified order in each iteration. Alternatively, we can employ a greedy and potentially more efficient method to construct the model as described in the following. We define two iteration loops: an outer iteration loop and an inner iteration loop. In each outer iteration, we estimate *μ *and . In the inner iterations, assuming *μ *and  are known and fixed, we estimate each *α_i _*and construct the model. Specifically, in each inner iteration, we first calculate each  from (8) and hen find , where *α_i _*stands for the value of *α_i _*obtained in the previous inner iteration. This step basically identifies the **x***_j _*that gives the largest increase in the log posterior distribution. Then we add, delete or retain **x***_j _*as described early. The inner iterations can run until a local convergence criterion is satisfied. Let vector  contain all finite *α_i_*s, vector  consist of the corresponding *β_i_*s and matrix  contain the corresponding columns of **X**. Then we essentially construct the following reduced model:(10)

where the number of columns of , *k_r_*, is typically much less than the number of rows, *n*. This property will be used to reduce computation.

To calculate  in (8), we need to first calculate *s_i _*and *q_i _*which requires . Since **C**_-*i *_is different for different *i*, it may need large computation to calculate all . However, it was shown in [16] that we can calculate *s_i _*and *q_i _*as follows(11)

where  and . This requires only one matrix inversion in each iteration for calculating all *s_i _*and *q_i_*, *i *= 1, ⋯, *k*. However, since **C **is a relatively large matrix of size *n *× *n*, direct calculation of **C**^-1 ^may still require large computation. To avoid this problem, we can use the Woodbury matrix identity to derive an expression for **C**^-1^:(12)

where(13)

with . The size of matrix **∑ **is *k_r _*× *k_r _*which is typically much smaller than the size of **C**. Since inverting a matrix of *N *× *N *using an efficient method such as QR decomposition needs computation of *O*(*N*^3^), calculating **∑ **requires much less computation than directly inverting **C**. Using (12), we can calculate *S_i _*and *Q_i _*as follows:(14)

So far we have derived the method for efficiently estimating *α*. Other two unknown parameters *μ *and  can be obtained by setting  and . This gives(15)

where **1 **is a vector whose elements are all 1, and [15](16)

where **∑***_ii _*is the *i*th diagonal element of **∑ **and(17)

After we get estimates of *μ*,  and ***α***, we finalize the model (10), where the prior distribution of  is now . For those **x***_i_*s not in the model, we can declare that they do not affect the quantitative trait because their regression coefficient is zero. For those **x***_i_*s in the matrix , the posterior distribution of their regression coefficients  is Gaussian with covariance **∑ **in (13) and mean **u **in (17) [15]. We can then use the z-score or more conservative *t*-statistics to test if  at certain significance level. In this paper, the posterior mean **u***_j _*of the *j*th effect is reported as the empirical Bayes estimate of *β_j_*, denoted by , and the posterior standard deviation, , is used as the standard error of .

We now summarize our fast EBLASSO algorithm as follows.

### Algorithm 1 (EBLASSO algorithm)

*1. Initialize parameters: choose a *> -1.5 *and b *> 0, *calculate μ *= **1***^T ^***y**/*n*, *and set **to be a small number, e.g., *.

*2. Initialize the model: Find , and calculate α_j _from *(9)*, set all other α_i_s to be ∞ and *.

*3. Calculate ***∑ ***from *(13)*, S_i _and Q_i_*, ∀*i, from *(14).

4. Update the model

   while the local convergence criterion is not satisfied

*      Calculate q_i _and s_i _from *(11), ∀*i*.

*      Calculate **from *(8), ∀*i*.

*   Find *.

*   if *

*      if ***x***_j _is in the model, delete it and update ***∑***, S_i _and Q_i_*, ∀*i*.

   else

*      if ***x***_j _is in the model, set **and update ***∑***, S_i _and Q_i_*, ∀*i*.

*      if ***x***_j _is not in the model, add it, set **and update ***∑***, S_i_*

*      and Q_i_*, ∀*i*.

   end if

   end while

*5*. *Update the residual variance **using *(16).

*6*. *Calculate ***∑ ***from *(13) *and ***C**^-1 ^*from *(12).

*7*. *Update the fixed effects μ using *(15) *and update *.

*8*. *Calculate S_i _and Q_i _from *(14).

*9*. *If the global convergence criterion is not satisfied, go to step 4*.

*10. Find the posterior mean of **from *(17) *and the posterior variance ***∑***_ii _from *(13).

*11*. *Use t-statistics to test if *.

The parameters *a *and *b *can be set to be a small number (e.g., *a *= *b *= 0.01) so that the Gamma prior distribution is essentially noninformative [10]. Alternatively, we can use the predicted error (PE) obtained from cross-validation [4] to choose the values of *a *and *b*. As done in [16], the initial value of  is chosen in step 1 to be a small number and the initial model is selected in step 2 to have a single effect that corresponds to the maximum *L*(*α_i_*) with *a *= -1. The outer iteration loop consists of steps 4-9, while the inner iteration loop is step 4, where we use the greedy method described earlier to update the model. In step 4, we use the method given in the Appendix of [16] to efficiently update **∑**, *S_i _*and *Q_i _*after a **x***_j _*is added to or deleted from the model or after *α_j _*is updated. The local convergence criterion can be defined as the simultaneous satisfaction of the following three conditions: 1) no effect can be added to or deleted from the model, 2) the change of *L*(***θ ***) between two consecutive inner iterations is smaller than a pre-specified small value, and 3) the change of  between two consecutive inner iterations is less than a pre-specified value. The global convergence criterion can be defined as the simultaneous satisfaction of the following two conditions: the change of *L*(***θ ***) between two consecutive outer iterations is smaller than a pre-specified small value, and 2) the total change in *μ*,  and  between two consecutive outer iterations is less than a pre-specified value. A Matlab program has been developed to implement the algorithm; and a more efficient C++ program is under development.

## Results

### Simulation study

We simulated a single large chromosome of 2400 centiMorgan (cM) long covered by evenly spaced *q *= 481 markers with a marker interval of 5 cM. The simulated population was an *F*_2 _family derived from the cross of two inbred lines with a sample size *n *= 1000. The genotype indicator variable for individual *i *at marker *k *is defines as *X_ik _*= 1, 0, -1 for the three genotypes, *A*_1_*A*_1_, *A*_1_*A*_2 _and *A*_2_*A*_2_, respectively. Twenty markers are QTLs with main effects and 20 out of the  marker pairs have interaction effects. The locations and effects of the markers and maker pairs are shown in Table 1. Environmental effects and *G *× *E *effects were not simulated. The true population mean is *μ *= 100 and the residual variance is .

**Table 1:** True and estimated QTL effects for the simulated data with main and epistatic effects.

Markers*^a^*	Position	True*^b^*	EBLASSO*^c^*	EB*^c^*
(*i, j*)	(cM, cM)	*β*(*h*^2^)		
(11,11)	(50,50)	4.47(0.0975)	4.5801(0.1612)	4.8593(0.2075)
(26,26)	(125,125)	3.16(0.0524)	3.0768(0.1576)	3.3221(0.2035)
(42,42)	(205,205)	-2.24(0.0250)	-2.3169(0.1796)	-2.2769(0.2262)
(48,48)	(235,235)	-1.58(0.0128)	-1.3171(0.1720)	-1.3634(0.2205)
(72,72)	(355,355)	2.24(0.0247)	-	1.6537(0.4277)
(73,73)	(360,360)	3.16(0.0506)	5.1247(0.1555)	3.8771(0.4219)
(123,123)	(610,610)	1.10(0.0062)	-	1.5168(0.2432)
(127,127)	(630,630)	-1.10(0.0063)	-	-1.1834(0.2460)
(161,161)	(800,800)	0.77(0.0030)	-	-
(181,181)	(900,900)	1.73(0.0152)	-	-
(182,182)	(905,905)	3.81(0.0725)	5.6744(0.2400)	5.5127(0.2894)
(185,185)	(920,920)	2.25(0.0263)	1.7123(0.2327)	1.7070(0.2858)
(221,221)	(1100,1100)	-1.30(0.0088)	-1.4276(0.1506)	-1.0867(0.1956)*^d^*
(243,243)	(1210,1210)	-1.00(0.0051)	-0.8603(0.1486)	-
(262,262)	(1305,1305)	-2.24(0.0245)	-2.2539(0.1826)	-1.6078(0.2417)
(268,268)	(1335,1335)	1.58(0.0120)	2.4264(0.2040)	2.1736(0.2509)
(270,270)	(1345,1345)	1.00(0.0049)	-	-
(274,274)	(1365,1365)	-1.73(0.0147)	-1.4114(0.1800)	-1.4935(0.2254)
(361,361)	(1800,1800)	0.71(0.0026)	0.7856(0.1457)	0.6520(0.1859)*^d^*
(461,461)	(2300,2300)	0.89(0.0040)	-	-
(5,6)	(20,25)	2.24(0.0230)	1.7839(0.1654)	1.5752(0.2886)
(6,39)	(25,190)	2.25(0.0128)	1.9691(0.2168)	-
(42,220)	(205,1095)	4.47(0.0511)	4.3836(0.2198)	4.6414(0.3394)
(75,431)	(370,2150)	0.77(0.0014)	1.1360(0.2124)*^d^*	-
(81,200)	(400,995)	-2.24(0.0128)	-2.4190(0.2460)	-
(82,193)	(405,960)	1.58(0.0063)	1.6345(0.2442)	-
(87,164)	(430,815)	3.16(0.0235)	2.9263(0.2254)	1.7059(0.3319)*^d^*
(87,322)	(430,1605)	3.81(0.0342)	4.1019(0.2274)	3.7040(0.3632)
(92,395)	(455,1970)	1.73(0.0081)	1.5714(0.2065)*^d^*	-
(104,328)	(515,1635)	1.00(0.0024)	0.8081(0.1979)*^d^*	-
(118,278)	(585,1385)	-2.24(0.0120)	-2.0796(0.2221)	-2.2590(0.3460)
(150,269)	(745,1340)	1.10(0.0028)	1.0740(0.2142)	-
(237,313)	(1180,1560)	0.71(0.0014)	-	-
(246,470)	(1225,2345)	-1.10(0.0032)	-1.2381(0.2114)*^d^*	-
(323,464)	(1610,2315)	0.89(0.0020)	-	-
(328,404)	(1635,2015)	-1.73(0.0079)	-2.3036(0.2123)	-1.9428(0.3330)
(342,420)	(1705,2095)	-1.30(0.0041)	-1.3886(0.2121)*^d^*	-
(344,407)	(1715,2030)	-1.00(0.0025)	-	-
(373,400)	(1860,1995)	-1.58(0.0070)	-1.4732(0.2028)	-
(431,439)	(2150,2190)	3.16(0.0278)	2.6700(0.2121)	2.2454(0.3366)*^d^*

*μ*		100	100.70	100.59
		10	11.76	0.25
CPU time			3.4 mins	249 hrs

*^a^*When *i *= *j*, the QTL is a main effect; otherwise, it is an epistatic effect.

*^b^*The true value of a QTL effect is denoted by *β *and the proportion of variance contributed by the QTL is denoted by *h*^2^.

*^c^*The estimated QTL effect is denoted by  and the standard error is denoted by . The EBLASSO algorithm used hyperparameters *a *= *b *= 0.1 and the EB algorithm used hyperparameters *τ *= -1 and *ω *= 0.001.

*^d^*The estimated QTL effect was obtained from a neighboring marker (≤ 20 cM away) rather than from the maker with the true effect.

The total phenotypic variance for the trait can be written as(18)

where cov(*x_j_*, *x*_*j*'_) is the covariance between *X_j _*and *X*_*j*' _if *j *≠ *j*' or the variance of *X_j _*if *j *= *j*', which can be estimated from the data. The total phenotypic variance was calculated from (18) to be  and the total genetic variance contributed by the main and epistatic effects of the markers was calculated from the second term of the right hand side of (18) to be 88.67. If we ignore the contributions from the covariance terms which are relatively small, the proportions of the phenotypic variance explained by a particular QTL effect *j *can be approximated by(19)

where var(*x_j_*) is the variance of *X_j_*. In the simulated data, the proportion of contribution from an individual QTL varied from 0.30% to 9.75%, whereas the proportion of contribution from a pair of QTLs ranged from 0.26% to 7.25%, as shown in Table 1. Some of the markers had only main or epistatic effect, while the others had both main and epistatic effects. The QTL model contained a total of  possible effects, a number about 115 times of the sample size.

The data were analyzed in Matlab on a personal computer (PC) using the EBLASSO algorithm, the EB method, the RVM and the LASSO. The Matlab program SPARSEBAYES for the RVM was downloaded from http://www.miketipping.com. We translated the original SAS program for the EB method [12] into Matlab, and slightly modified the program to avoid possible memory overflow due to the large number of possible effects. We also got the program glmnet [19] that is a very efficient program implementing the LASSO and other related algorithms. The PC version of glmnet uses Matlab to initialize and call the core LASSO algorithm that is implemented efficiently with Fortran code.

**Figure 1:**
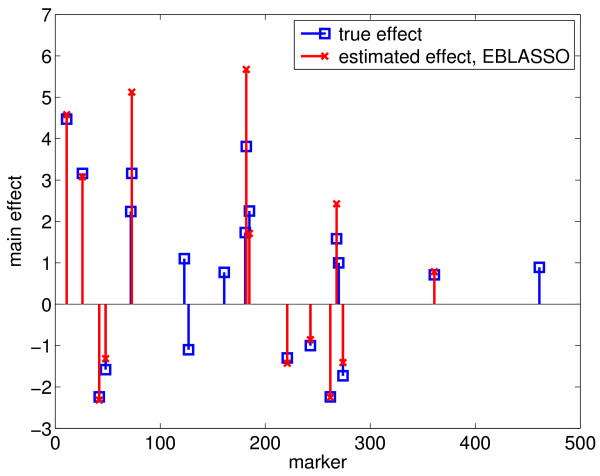
**Main effects estimated with the EBLASSO for the simulated data with main and epistatic effects**.

**Figure 2:**
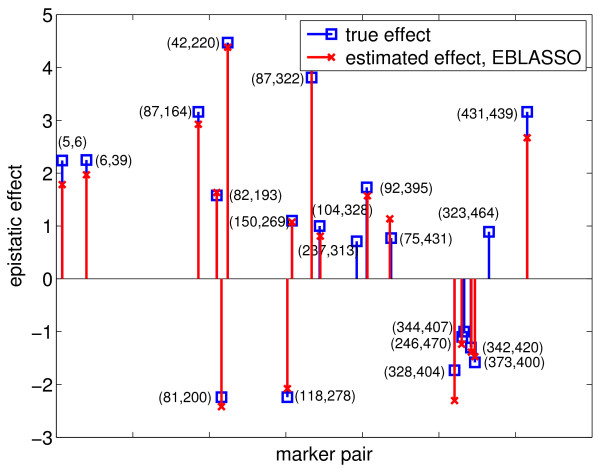
**Epistatic effects estimated with the EBLASSO for the simulated data with main and epistatic effects**. The horizontal axis is scaled as  for each marker pair (marker *i*, marker *j*).

We used the PE [4] obtained from ten-fold cross validation to select the values of hyperparameters *a *and *b *in our EBLASSO algorithm. Ideally, we should test a large set of values for *a *≥ -1 and *b *> 0, but this may be time consuming. Therefore, we first ran cross-validation for the following values: *a *= *b *= 0.001, 0.01, 0.05, 0.1, 0.5, 1; the degree of shrinkage generally decreases along this path. Table 2 lists the PEs and the standard errors of the PEs for different values of *a *and *b*. It is seen that the PE for *a *= *b *= 0.1 is the smallest, although it is close to the PEs for *a *= *b *= 0.05 and 0.5 but relatively smaller than PEs for the other values of *a *and *b*. To see if *a *= *b *= 0.1 is the best set of values, we further ran cross-validation with *b *= 0.1 and *a *= 0.5 or -0.01. For a fixed *b*, the degree of shrinkage decreases when *a *decreases. It is seen from Table 2 that the PE for *b *= 0.1 and *a *= 0.5 or -0.01 is greater than that for *a *= *b *= 0.1. Therefore, cross-validation gave *a *= *b *= 0.1 as the best set of values. Table 2 also lists the number of effects detected with different values of *a *and *b*. All 30 effects detected with *a *= *b *= 0.1 are presented in Table 1 and shown in Figures 1 and 2.

**Table 2:** Summary of results for the simulated data with main and epistatic effects

Algorithm	Parameters^◇^	PE ± STE*	Number of effects^†‡^		CPU time (mins)
	(0.001, 0.001)	16.49 ± 0.8908	25/0	13.5	3.4
	(0.01, 0.01)	15.95 ± 0.7477	28/0	12.46	3.4
	(0.05, 0.05)	15.89 ± 0.7498	30/0	11.72	3.4
	(0.1, 0.1)	15.81 ± 0.8359	30/0	11.72	3.4
EBLASSO	(0.5, 0.5)	15.86 ± 0.7717	31/0	11.57	3.4
	(1, 1)	16.07 ± 0.7203	29/0	12.31	3.4
	(0.5, 0.1)	16.14 ± 0.8557	28/0	12.5	3.4
	(-0.01, 0.1)	15.92 ± 1.0161	32/1	11.31	3.4

	(-1, 0.0001)	-	14/1	21.22	2,760.0
EB	(-1, 0.0005)	-	13/1	12.15	4,140.0
	(-1, 0.001)	-	22/1	0.25	14,940.0
	(-1, 0.01)	-	8/0	0.01	2,760.0

^◇ ^Parameters are (*a, b*) for the EBLASSO, and (*τ*, *ω*) for the EB.

*The average PE and the standard error were obtained from ten-fold cross validation.

^†^The number of effects and residual variance were obtained using all 1000 samples not from cross validation.

^‡^The first number is the number of true positive effects; the second number is number of false positive effects. All the effects counted have a p-value ≤ 0.05.

**Figure 3:**
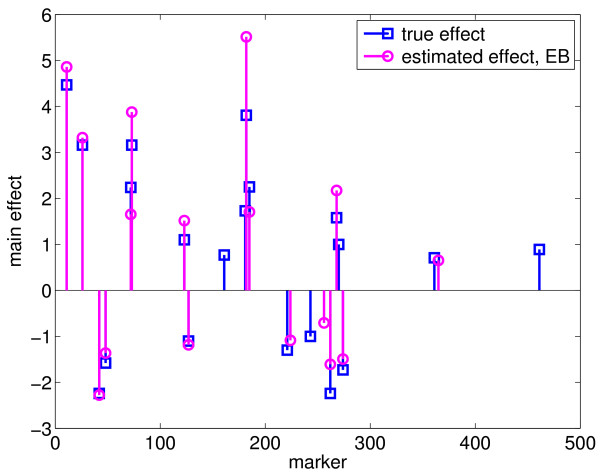
**Main effects estimated with the EB method for the simulated data with main and epistatic effects**.

**Figure 4:**
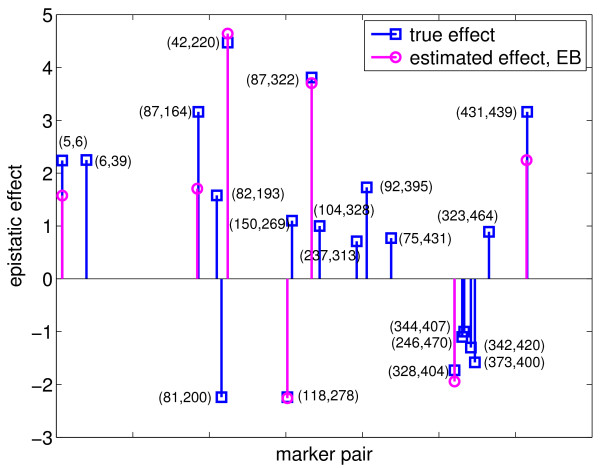
**Epistatic effects estimated with the EB method for the simulated data with main and epistatic effects**. The horizontal axis is scaled  for each marker pair (marker *i*, marker *j*).

To test the performance of the EB method, we ran the program with the following parameter values: *τ *= -1, *ω *= 0.0001, 0.0005, 0.001 and 0.01, which yielded 14, 13, 22, and 8 true simulated effects, respectively, and 1, 1, 1, and 0 false effects, respectively, as shown in Table 2. Cross-validation was not done because it was too time-consuming, and thus, the optimal values for the parameters could not be determined. Nevertheless, we listed 22 true positive effects estimated with *τ *= -1 and *ω *= 0.001 in Table 1, which reflects the best performance of the EB method with the set of parameters values tested. We also plotted these 22 effects in Figures 3 and 4. Comparing the effects detected by EBLASSO and EB methods shown in Table 1 and in Figures 1, 2, 3 and 4, the EBLASSO method detected 13 (17) true main (epistatic) effects, whereas the EB method detected 15 (7) true main (epistatic) effects. Overall, the EBLASSO detected 8 more true effects than the EB method without any false positive effects, whereas the EB method gave one false positive effect. We would like to emphasize here that the EB method may detect less number of true effects in practice because as we mentioned earlier it is too time-consuming to choose the optimal values for the parameters *τ *and *ω*. To see if EBLASSO could estimate QTL effects robustly, we simulated three replicates of the data: each replicate consists of 1000 individuals whose genotypes at 481 markers were independently generated and whose phenotypes were calculated from (2) with **e **independently generated from Gaussian random variables with zero mean and covariance 10**I**. We performed cross-validation and determined the best values of *a *and *b *for each replicate. Using these values, we ran the EBLASSO and identified 35, 38, 34 true positive effects and 4, 6, 2 false positive effects, respectively, for three replicates. These results showed that the EBLASSO could detect QTL effects robustly.

The EBLASSO took about 3.4 minutes of CPU time for each set of values of *a *and *b *listed in Table 2, whereas the EB took 249 hours of CPU time for *τ *= -1 and *ω *= 0.001 and about 46, 69, 46 hours for *τ *= -1 and *ω *= 0.0001, 0.0005, 0.01, respectively. This simulation study showed that the EBLASSO method not only can detect more effects, but also offers a huge advantage in terms of computational time. Note that all simulations were done in Matlab. It is expected that the EBLASSO algorithm will be even faster, after its implementation in C++ is completed.

We wished to test the performance of the RVM and the LASSO on the simulated data. To this end, we replaced the inner iteration in our EBLASSO algorithm with the program SPARSEBAYES that implemented the RVM. Although we carefully modified SPARSEBAYES to avoid possible memory overflow due to the large number of possible effects, the program ran out of memory after one or two outer iterations. Hence, we did not get any results from the RVM method. Considering the QTL model in (2), the LASSO tries to estimate *μ *and the QTL effects ***β ***as follows(20)

where *λ *is a positive constant that can be determined with cross-validation [4]. We tried to run the program glmnet [19] with the simulated data. However, glmnet could not handle the big design matrix **X **of 1, 000 × 115, 922 in our QTL model, and we did not get any results from glmnet.

In order to compare the performance of our EBLASSO algorithm with that of the RVM and the LASSO, we simulated a new set of data by deleting the 20 epistatic effects in the previous set of simulated data, and then used a QTL model that only contained 481 possible main effects to estimate QTL effects, i.e., the design matrix **X **in (2) was 1000 × 481. The small number of possible effects was chosen to avoid the memory overflow problem of the RVM and glmnet. The results of the EBLASSO, EB, RVM and LASSO for this data set are summarized in Table 3. To choose the values of *a *and *b *for the EBLASSO, we ran ten-fold cross validation with the following parameters: *a *= *b *= 0.001, 0.01, 0.05, 0.1, 0.5, 1. Since *a *= *b *= 0.1 yielded the smallest PE, we further performed cross-validation with *b *= 1, and *a *= -0.95, -0.75, -0.5, 0.5. This gave the smallest PE at *a *= -0.75 and *b *= 0.1. Finally, we used *a *= -0.75 and *b *= 0.1 to run the EBLASSO algorithm on the whole data set, which identified all 20 true effects but also 5 false positive effects. In fact, since the EBLASSO program ran very fast on this data set, we did ten-fold cross validation for 165 sets of values for *a *and *b *obtained by combining the following values: *a *= -1, -0.95, -0.85, -0.75, -0.5, -0.1, -0.05, -0.01, -0.001, 0.001, 0.01, 0.05, 0.1, 0.5, 1 and *b *= 0.001 0.01 0.05 0.1, 0.5, 1, 2.5, 5, 10, 15, 20. The smallest average PE and standard error were 10.49 ± 0.5444 achieved at *a *= 0.05 and *b *= 10. With this set of values, the EBLASSO identified all 20 true effects and also 6 false positive effects, which is similar to that identified at *a *= -0.75 and *b *= 0.1. This study showed that the two-step cross-validation approach, where cross-validation was run with *a *= *b *= 0.001, 0.01, 0.05, 0.1, 0.5, 1 and then with a fixed *b *but a varying *a*, performed well and could save much time. We further simulated three replicates of the data in the same way described earlier for the model with epistatic effects. We then repeated the two-step cross-validation for each replicate. Using the values of *a *and *b *determined in cross-validation, the EBLASSO detected 20, 19, 19 true effects, and 4, 1 and 2 false positive effects, respectively, for three replicates. This again showed that our EBLASSO could estimate the effects robustly.

**Table 3:** Summary of results for the simulated data with only main effects

Algorithm	Parameters^◇^	PE ± STE*	Number of effects^†‡^		CPU time (sec)
	(0.001, 0.001)	11.52 ± 0.5677	14/0	11.1	1.2
	(0.01, 0.01)	11.52 ± 0.578	16/0	10.53	1.3
	(0.05, 0.05)	11.36 ± 0.6088	17/0	10.32	1.1
	(0.1, 0.1)	11.23 ± 0.5571	17/0	10.32	1.1
	(0.5, 0.5)	11.32 ± 0.5937	17/0	10.34	1.1
EBLASSO	(1, 1)	11.4 ± 0.5929	16/0	10.64	1.1
	(0.5, 0.1)	11.57 ± 0.5593	15/0	10.83	1.3
	(-0.5, 0.1)	10.87 ± 0.5599	17/0	10.31	1.6
	(-0.75, 0.1)	10.78 ± 0.5646	20/5	9.52	1.5
	(-0.95, 0.1)	11.09 ± 0.5045	22/20	8.71	1.4
	(-1, 0.0001)	17.73 ± 2.0244	9/0	16.07	1491.9

	(-1, 0.0005)	15.81 ± 2.5732	16/0	11.66	1676.0
EB	(-1, 0.001)	12.21 ± 1.7635	17/2	10.65	1657.9
	(-1, 0.01)	10.69 ± 0.9903	19/4	9.05	1954.9
	(-1, 0.1)	11.63 ± 0.5743	20/20	7.29	2222.7

RVM	-	-	20/42	7.81	268.7

	0.1347	10.77 ± 0.4583	16/27	9.47	0.7
	0.0850	10.52 ± 0.4442	20/49	8.89	0.7
LASSO	0.0675	10.50 ± 0.5248	20/48	8.63	0.7
	0.0536	10.52 ± 0.4382	19/35	8.28	0.7
	0.0338	10.59 ± 0.4434	17/2	7.35	0.7

^◇ ^Parameters are (*a, b*) for the EBLASSO, (*τ*, *ω*) for the EB and *λ *for the LASSO.

*The average PE and the standard error were obtained from ten-fold cross validation.

^†^The number of detected effects and residual variance were obtained using all 1000 samples not from cross validation.

^‡^The first number is the number of true positive effects; the second number is number of false positive effects. All the effects counted have a p-value ≤ 0.05.

The optimal values for the parameters of the EB method were *τ *= -1 and *ω *= 0.01, since they gave the smallest PE in cross-validation as listed in Table 3. With *τ *= -1 and *ω *= 0.01, the EB method detected 19 true effects and 4 false positive effects. The RVM detected all 20 true effects as the EBLASSO did, but it also output a large number of 42 false positive effects. This result is consistent the observation [13] that the uniform prior distribution used in the RVM usually yields many false positive effects. To choose the optimal value of *λ *for the LASSO, we ran ten-fold cross validation starting from *λ *= 4.9725 (which gave only one nonzero effect) and then decreasing *λ *to 0.0025 with a step size of 0.0768 on the logarithmic scale (Δ ln(*λ*) = 0.0768). The smallest PE was achieved at *λ *= 0.0675. We then used this value to run glmnet on the whole data set, which yielded 97 nonzero effects. For each of these nonzero effects, we calculated their standard error using equation (7) in [4], and then calculated the p-value of each nonzero effect. This gave 20 true effects and 48 false positive effects with a p-value less than 0.05. Comparing the number of effects detected by the EBLASSO, EB, RVM and LASSO, the EBLASSO offered the best performance because it detected all true effects and a very small number of false positive effects.

It is seen from Table 3 that the EBLASSO and the LASSO took much less time than the EB method and the RVM on analyzing this data set. It is expected that the EBLASSO is much faster than the EB method because as we discussed earlier the EB needs a numerical optimization procedure. The RVM and EBLASSO generally should have similar speed because two algorithms use the similar technique to estimate effects. However, when applying to the same data set, the RVM often yields a model with much more nonzero effects than the EBLASSO as is the case here, because the RVM does not provide sufficient degree of shrinkage. Due to this reason, the RVM algorithm requires more time than the EBLASSO. The LASSO took slightly less CPU time than the EBLASSO in this example. However, we would emphasize that the LASSO was implemented with Fortran but our EBLASSO was implemented with Matlab. The speed of EBLASSO is expected to increase significantly once it is implemented in C/C++.

### Real data analysis

This dataset was obtained from [20]. This dataset consists of *n *= 150 double haploids (DH) derived from the cross of two spring barley varieties Morex and Steptoe. The total number of markers was *q *= 495 distributed along seven pairs of chromosomes of the barley genome, covering 206 cM of the barley genome. The phenotype was the spot blotch resistance measured as the lesion size on the leaves of barley seedlings. Note that spot blotch is a fungus named *Cochliobolus sativus*. This dataset was used as an example for the application of the EBLASSO method. Genotype of the markers were encoded as +1 for genotype A (the Morex parent), -1 for genotype B (the Steptoe parent), and 0 for missing genotype. Ideally, the missing genotypes should be imputed from known genotypes of neighboring markers. For simplicity, we replaced the missing genotypes with 0 in order to use the phenotypes of the individuals with missing genotypes. The total missing genotypes only account for about 4.2% of all the genotypes. Including the population mean, the main and the pair-wise epistatic effects, the total number of model effects was , about 818 times as large as the sample size.

**Figure 5:**
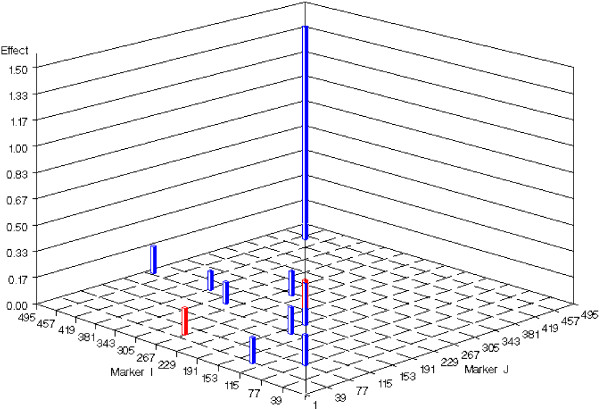
**Effects estimated with the EBLASSO algorithm for the real data**. Blue bars represent the positive effects, while the red bars represent the absolute values of negative effects.

Table 4 gives the average PE and the standard error obtained from 5-fold cross validation, the residual variance and the number of effects detected by the EBLASSO method for different values of *a *and *b*. It is seen that the PEs for *a *= *b *= 0.001, 0.01.0.05 are almost the same but are smaller than the PEs for other larger *a *and *b*. However, when *a *= *b *= 0.001 or 0.01, only one or two effects were detected. When *a *= *b *= 0.1, 0.5 or 1, the residual variance is very small, implying that the model is likely over-fitted. Specifically, the number of columns of matrix  in the model (10) is equal to the total number nonzero effects, which is more than 120 for *a *= *b *= 0.1, 0.5 or 1 as indicated in Table 4. Hence, since the number of samples (150) is relative small, **y **- *μ *can be almost completely in the column space of , which results in very small residual variance. Based on these observations, it seems that *a *= *b *= 0.05 gives reasonable results, because the PE is among the smallest and the residual variance is relatively but not unreasonably small. Nevertheless, in order to estimate effects more reliably, we searched over all effects detected with *a *= *b *= 0.05, 0.07, 0.1, 0.5 or 1, and found eight effects are detected with all these values. Markers or marker pairs of these eight effects and their values estimated with *a *= *b *= 0.05 were listed in Table 5. All 11 effects estimated with *a *= *b *= 0.05 were also plotted in Figure 5. As seen from Table 5, one single QTL at marker 446 contributes most of the phenotypic variance (about 76%), while the other QTL effects contribute from about 0.8% to 2.6% of the variance. We also analyzed the data with the EB method. One effect at marker 446 was detected with a p-value < 0.05 when *τ *= -1, *ω *= 0.0001 or 0.0005, no effect was detected with p-value ≤ 0.05 when *τ *= -1, *ω *= 0.001 or 0.01. The CPU time of the EB was about 46 minutes for each set of values of *τ *and *ω *tested; whereas the CPU time of the EBLASSO method was about 3 minutes for *a *= *b *= 0.05.

**Table 4:** Summary of the results of the EBLASSO algorithm for the real data

*a *= *b*	PE ± STE*	Number of effects^†‡^	
0.001	0.70 ± 0.21	1/1/1	0.6706
0.01	0.79 ± 0.31	2/2/2	0.5996
0.05	0.70 ± 0.21	11/11/11	0.2699
0.07	0.96 ± 0.30	10/15/15	0.2104
0.1	1.20 ± 0.18	13/128/132	2.59E-06
0.5	1.21 ± 0.09	9/112/122	2.59E-06
1	1.25 ± 0.17	8/115/132	2.59E-06

*The average PE and the standard error were obtained from five-fold cross validation.

^†^The number of effects and residual variance were obtained using all 150 samples not from cross validation.

^‡^The first number is the number of effects with a p-value ≤ 0.05 and proportion of variance ≥ 0.5%; the second number is the number of effects with a p-value ≤ 0.05; the third number is the total number of non-zero effects reported by the program.

**Table 5:** Eight effects estimated with the EBLASSO algorithm for the real data.

Markers		
(446,446)	1.4173(0.0432)	0.7639
(187,187)	0.2624(0.0421)	0.0262
(77,77)	0.1881(0.0413)	0.0132
(238,238)	-0.1742(0.0427)	0.0101
(197,483)	0.1748(0.0405)	0.0117
(37,130)	0.1557(0.0422)	0.0085
(53,270)	-0.1649(0.0452)	0.0081
(149,175)	0.1697(0.0464)	0.0078

	5.7037	
	0.2699	
		0.8495

Eights effects were detected with all of the following parameters: *a *= *b *= 0.05, 0.07, 0.1, 0.5, 1; ,  and  listed here were obtained with *a *= *b *= 0.05.

## Discussion

Xu [12] compared several methods for multiple QTL mapping including the EB [12], LASSO [4], penalized likelihood (PENAL) [21] and stochastic search variable selection (SSVS) [22,23] methods. The SSVS method is much slower than the EB method; whereas LASSO and PENAL methods are faster than the EB method. Although we did not directly compare the speed of our EBLASSO with that of the PENAL method, based on all comparisons with the EB method in [12], we observed that the EBLASSO method is faster than PENAL methods. Direct comparison between the EBLASSO and LASSO showed that the LASSO is slightly faster than the current version of EBLASSO, however, this may not be the case when the EBLASSO is implemented in C/C++ instead of Matlab. Although EB, LASSO, PENAL and SSVS methods all produced satisfactory results in a simulation study [12], the EB method outperformed the other three methods in terms of the mean-squared error. Moreover, when being applied to a real data set, the EB and LASSO detected some effects, whereas the PENAL and SSVS failed to generate any meaningful results [12]. In our simulation studies, we observed that the EBLASSO method detected more true effects than the EB method with almost the same false positive rate, and the same number of true effects as the LASSO but with a much smaller number of false positive effects. When analyzing a real data set, we found that the EBLASSO method detected a reasonable number of effects, but the EB method detected one or zero effect depending on values of the hyperparameters used. These observations in both simulation study and real data analysis demonstrated that the EBLASSO method outperforms the EB method and the LASSO.

The EBLASSO method was built upon the idea of the RVM in machine learning. The EBLASSO and EB methods, as well as the RVM, all are based on a Bayesian hierarchical linear regression model and all estimate the variances of the regression coefficients. The difference of the three methods in the regression model is the different prior distributions for the hyperparameters. The EB method and the RVM employ inverse chi-square and uniform distributions, respectively, for the variances of the regression coefficients, while the EBLASSO assigns exponential distributions to the variance components and uses a Gamma distribution for the parameter of the exponential distribution, which leads to the prior distribution in (3) for the variance components. The uniform prior distribution used by the RVM may not provide enough degree of shrinkage for certain data and thus generate a large number of false positive effects as shown in [13] and as demonstrated in our simulation study.

The prior distributions used by the EBLASSO and RVM methods enable one to estimate the variance components in a closed form, while the EB method generally needs a numerical optimization algorithm to estimate the variance components. This difference has at least two implications: 1) both the EBLASSO and the RVM methods requires much less computation than the EB method to estimate the variance components, and 2) the EBLASSO method and the RVM method can always find the unique optimal estimate of a variance component but the numerical optimization algorithm used by the EB method may not find the optimal value of the variance due to the nonlinearity and non-convexity of the objective function. Another main factor that makes the EBLASSO method and RVM more efficient is an automatic variable selection procedure resulting from the process of estimating variance components, because the variables whose precision is infinity or equivalently whose variance is zero are excluded from the model. This results in an efficient formula in (12) for calculating the inverse of the covariance matrix of the data. This is especially beneficial when the number of samples is relatively large. On the other hand, the EB method in principle can be applied to a linear regression model with any prior distribution for the variances of regression coefficients. Since the prior distribution may play an important role in estimation of the QTL effects, the EB method has its value when one tries to explore different prior distributions.

To get the best performance, the EBLASSO method needs to properly choose values of hyperparameters *a *and *b*. In this paper, we selected the values of *a *and *b *that gave the smallest average PE resulting from ten-fold cross validation. Ideally, we need to find PEs for a large set of values for *a *≥ -1 and *b *> 0 and then identify the best values for *a *and *b*. In our simulation study, we found a two-step cross validation procedure could significantly reduce the number of values to be tested without missing the best values, thereby reducing computational time. In this two-step procedure, we first run cross-validation for the following set of values: *a *= *b *= 0.001, 0.01, 0.05, 0.1, 0.5, 1. We identify the values (denoted as a* and b*) from this set of values that yields the smallest PE. We then fixed *b *to be b* and run cross-validation for several other values of a greater or less than a*. The final best values of *a *and *b *are the ones that yield the smallest PE.

The EBLASSO algorithm may still be improved. In the analysis of simulated data with both main and epistatic effects, although the EBLASSO method detected 8 more true effects than the EB method without any false positive effects, it missed three effects that the EB method detected. It is unclear how this discrepancy occurred. One possible reason is the different prior distributions used in the two methods. Although it is difficult for the EBLASSO method to use the scaled inverse chi-square distribution that is used by the EB method, other prior distributions may worth investigation. Another possible reason may be the greedy method used to select the variable to include in or to exclude from the model. In the current algorithm, we choose the variable that gives the largest increase in the likelihood to add to or delete from the model. It may be better to simultaneously add or delete more than one variables. The EBLASSO method presented in this paper assumes continuous quantitative traits. It can also be extended to handle binary or polychotomous traits and the algorithm is under development. The algorithm is currently implemented in Matlab. We are developing programs in C++ to implement the algorithm, which is expected to be much faster and to be capable of running in R and SAS environments.

## Conclusions

We have developed a fast empirical Bayesian LASSO method for multiple QTL mapping that can deal with a large number of effects possibly including main and epistatic QTL effects, environmental effects and the effects of environment and gene interactions. Our simulation studies demonstrated that the EBLASSO algorithm needed about 3.4 minutes of CPU time, running in Matlab on a PC with 2.4 GHz Intel Core2 CPU and 2 Gb memory running Windows XP, to analyze a QTL model with more than 10^5 ^possible effects, whereas the EB method took more than 2,000 minutes to analyze the same model on the same computer. Our simulation studies also showed that the EBLASSO method could detect more true effects with almost the same false positive rate comparing to the EB method. Our real data analysis demonstrated that the EBLASSO method could output more reasonable effects than the EB method. Comparing with the LASSO, our simulation showed that the current version of the EBLASSO implemented in Matlab was slightly slower than the LASSO implemented with glmnet in Fortran, and that the EBLASSO detected the same number of true effects as the LASSO but a much smaller number of false positive effects. In conclusion, the EBLASSO method will be a useful tool in multiple QTL mapping.

## Authors' contributions

XC conceived the idea of the EBLASSO method, developed the EBLASSO algorithm, participated in the data analysis and drafted the manuscript. AH developed the computer programs and performed the data analysis. SX participated in the development of the EBLASSO algorithm, designed simulation study, participated in the data analysis and helped to draft the manuscript. All authors read and approved the final manuscript.
